# Can aging be programmed? A critical literature review

**DOI:** 10.1111/acel.12510

**Published:** 2016-08-17

**Authors:** Axel Kowald, Thomas B. L. Kirkwood

**Affiliations:** ^1^ Institute of Cell and Molecular Biosciences, and Institute for Ageing Newcastle University Campus for Ageing and Vitality Newcastle upon Tyne NE4 5PL UK; ^2^ Center for Healthy Aging Department of Cellular and Molecular Medicine University of Copenhagen Blegdamsvej 3B 2200 Copenhagen Denmark

**Keywords:** computer simulation, declining selection pressure, evolution of aging, kin selection, programmed aging

## Abstract

The evolution of the aging process has long been a biological riddle, because it is difficult to explain the evolution of a trait that has apparently no benefit to the individual. Over 60 years ago, Medawar realized that the force of natural selection declines with chronological age because of unavoidable environmental risks. This forms the basis of the mainstream view that aging arises as a consequence of a declining selection pressure to maintain the physiological functioning of living beings forever. Over recent years, however, a number of articles have appeared that nevertheless propose the existence of specific aging genes; that is, that the aging process is genetically programmed. If this view were correct, it would have serious implications for experiments to understand and postpone aging. Therefore, we studied in detail various specific proposals why aging should be programmed. We find that not a single one withstands close scrutiny of its assumptions or simulation results. Nonprogrammed aging theories based on the insight of Medawar (as further developed by Hamilton and Charlesworth) are still the best explanation for the evolution of the aging process. We hope that this analysis helps to clarify the problems associated with the idea of programmed aging.

## Introduction

Many people, coming new to the question of why and how aging occurs, are attracted naturally to the idea of a genetic programme. Aging is necessary, it is suggested, either as a means to prevent overcrowding of the species’ environment or to promote evolutionary change by accelerating the turnover of generations. The idea that aging is a programmed trait that is beneficial for the species, was first articulated by Weismann (1891), but is now generally accepted to be wrong (Kirkwood & Melov, 2011; de Grey, 2015), because it relies on group selection, which normally is much weaker than selection at the level of the individual (Maynard Smith, 1976), and it is circular in the way that it assumes that older individuals who do not age are generally worn out (Medawar, 1952).

Instead of programmed aging, the explanation for why aging occurs is thought to be found among three ideas all based on the principle that within iteroparous species (those that reproduce repeatedly, as opposed to semelparous species, where reproduction occurs in a single bout soon followed by death), the force of natural selection declines throughout the adult lifespan (Medawar, 1952). This decline occurs because at progressively older ages, the fraction of the total expected reproductive output that remains in future, on which selection can act to discriminate between fitter and less‐fit genotypes, becomes progressively smaller. The *mutation accumulation theory* (Medawar, 1952) assumes that over evolutionary time, there is a constant generation of deleterious mutations that are only expressed beyond a certain age. Natural selection generally favours the elimination of deleterious genes, but if its force is weakened by age, and because fresh mutations are continuously generated (Crow & Kimura, 1970), a mutation–selection balance results, which is (for dominant mutations) given by μ/s, where μ is the mutation rate and *s* is the selection disadvantage. The *antagonistic pleiotropy theory* (Williams (1957) suggests that a gene that has a benefit early in life, but is detrimental at later stages of the lifespan, can overall have a net positive effect and will be actively selected. Possible examples of antagonistic effects are a high testosterone level that is good for increased reproduction but might increase the late life risk of prostate cancer (Gann *et al*., 1996), or the deactivation of telomerase, which might protect against cancer but also leads to cellular senescence (Wright & Shay, 1995). The *disposable soma theory* (Kirkwood, 1977; Kirkwood & Rose, 1991) is concerned with optimizing the allocation of resources between maintenance on the one hand and other processes such as growth and reproduction on the other hand. An organism that invests a larger fraction of its energy budget in preventing accumulation of damage to its proteins, cells and organs will have a slower rate of aging, but it will also have fewer resources available for growth and reproduction, and *vice versa*. Mathematical models of this concept show that the optimal investment in maintenance (which maximizes fitness) is always below the fraction that is necessary to prevent aging (Kirkwood & Rose, 1991; Drenos & Kirkwood, 2005).

One argument against programmed aging was the view, generally accepted for a long time (Medawar, 1952; Lack, 1954; Berry & Bronson, 1992), that in the wild only a negligible fraction of a population survives long enough to die because of aging‐related mortality. Absence of significant senescence in the wild would speak against the evolution of a program for aging both by removing any potential advantage of actively destroying aged individuals (which would not normally be seen) and by making it hard to see how a program to drive a process not actually realized could have occurred. However, recent field studies have provided solid evidence that aging is a phenomenon that can also be seen in wild populations of a wide range of species (Brunet‐Rossinni & Austad, 2006; Bouwhuis *et al*., 2012; Nussey *et al*., 2013). Of course, rarity of observing aging in the wild could never have been absolute, because even for the nonprogrammed theories the elaborate arsenal of longevity assurance mechanisms can only have evolved if there was enough age‐related mortality in the wild to generate the required selection pressure. This would have been the case particularly for the evolution of increased longevity, which is thought often to have resulted following adaptations to reduce extrinsic mortality (e.g. by evolving flight as in birds and bats), which would have left populations to experience increased exposure to intrinsic deterioration (senescence) until secondary adaptations increasing longevity assurance occurred. Nevertheless, the new appreciation that aging in the wild is widespread has lessened the force of one of the traditional arguments against programmed aging, at least allowing for its theoretical possibility. Therefore, it is all the more important to inspect specific versions of programmed aging theories and explain why they are flawed.

This debate is not only of theoretical interest but has practical implications for the types of experiments that are performed to examine the mechanistic basis of aging (Kirkwood & Melov, 2011). If there is a genetic programme for aging, there would be genes with the specific function to impair the functioning of the organism, that is to make it old. Under those circumstances, experiments could be designed to identify and inhibit these genes, and hence to modify or even abolish the aging process. However, if aging is nonprogrammed, the situation would be different; the search for genes that actively cause aging would be a waste of effort and it would be too easy to misinterpret the changes in gene expression that occur with aging as primary drivers of the senescent phenotype rather than secondary responses (e.g. responses to molecular and cellular defects). It is evident, of course, that genes influence longevity, but the nature of the relevant genes will be very different according to whether aging is itself programmed or not.

Despite the cogent arguments that aging is not programmed, efforts continue to be made to establish the case for programmed aging, with apparent backing from quantitative models. It is important to take such claims seriously, because challenge to the existing orthodoxy is the path by which science often makes progress. However, it is important also to look closely at such claims, because the same rigour needs to be applied to checking the accuracy and validity of a model as for an experiment. Within this paper, we undertake a critical evaluation of the models that have been proposed to support programmed aging. In each case, we identify significant faults that undermine the conclusions drawn by the authors. It will be seen that the models that need to be considered have relied extensively on simulation techniques rather than on mathematical analysis. While analytical (mathematical) models generally have the advantage of clarity, they quickly become intractable when the phenomenon to be analysed depends on features such as spatial effects, which are at the heart of several of the claims made in favour of programmed aging. Therefore, a large part of this study is based on computer simulations of theoretical models because we have needed to assess the models within their own terms. We believe that access to the necessary computer code is important to recreate simulations and look for underlying assumptions that might not be explicitly mentioned in the publications. Therefore, the source code as well as executable versions of the programs is available as supplemental material (Data S1–S6).

## Lifespan controllability

**Figure 1:**
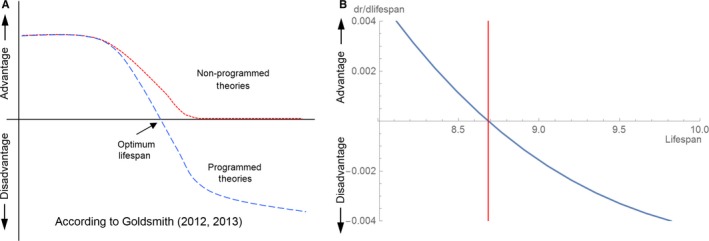
(A) Selective force for lifespan extension as a function of current lifespan (redrawn from Goldsmith (2013)). (B) Selective force for lifespan extension as a function of current mean lifespan according to the disposable soma theory (Kirkwood & Rose, 1991). The vertical line indicates the optimal lifespan, at which the selective forces to change lifespan become zero. Selective force is expressed as the infinitesimal change of Malthusian fitness (*r*) for an infinitesimal change of lifespan. Used parameter values: μ_min_ = 0.02, β_0_ = 0.25, γ = 0.1, *a*_min_ = 2, *h*_max_ = 1.5, *V* = 0.2 and *s*′ = 0.8.

Longevity varies not only between species, but is sometimes also affected by environmental and genetic factors within a species. Theories on evolution of aging need, therefore, to be able to explain the controllability of the lifespan. Goldsmith (2012, 2013) argued that the ability of an organism to fine tune its lifespan in response to temporary changes of the environment (e.g. caloric restriction) is incompatible with nonprogrammed theories and therefore speaks for programming. In both publications, he referred to a diagram showing the selection pressure to increase lifespan as a function of the current lifespan (Fig. 1A). According to Goldsmith, only programmed theories can produce a curve that crosses the abscissa (like the dashed line). Such a pattern indicates that there is a lifespan below which there is a selection pressure to increase lifespan and above which there is selection pressure to shorten lifespan. However, this claim is mistaken. Both the disposable soma and antagonistic pleiotropy theories are based on trade‐offs and thus produce exactly this behaviour. To illustrate this point, Fig. 1B shows an explicit calculation of the disposable soma theory with parameter values that result in an optimal average lifespan at 8.68. If the organism were to invest more in maintenance, it would increase its lifespan, but this would reduce evolutionary fitness (because fertility is reduced) as measured by the Malthusian parameter, *r* (Stearns, 1992). Consequently, there would be selection pressure to invest less in maintenance, which automatically restores the lifespan to the optimum.

The mutation accumulation theory, however, is not based on a trade‐off and indeed generates a curve such as the dotted line in Fig. 1A. Goldsmith (2012) states *non‐programmed theories of aging depend on the idea that the net (of any tradeoffs) evolutionary force toward living and reproducing beyond some species‐specific age is effectively zero*. He seems to imply that a value even slightly above zero would lead to a constantly increasing lifespan. This is again mistaken. As explained above, over evolutionary time scales, there is a constant generation of deleterious genes, which accumulate in the population to a level that is controlled by mutation–selection balance, given by μ/s. And because the mutation accumulation theory assumes that the expression of deleterious genes is age specific, their steady‐state level increases with age (because ‘s’ decreases with age). So the net effect of the two selective forces – (i) those that increase mortality due to deleterious genes and (ii) those that increase lifespan because that would increase fitness – can result in an increase in age‐specific mortality despite a remaining small selection pressure to increase lifespan. This is the whole point of the mutation accumulation theory and can also be shown mathematically (Charlesworth, 2001).

Finally, Goldsmith seems to think that only programmed aging theories allow for a fine tuning of the lifespan by the organism, for example, in response to environmental variation. Obviously, such a feature could be adaptive, and the life‐extending effect of caloric restriction has been suggested to result from such evolved plasticity (Harrison & Archer, 1989; Holliday, 1989). Analysis by Shanley & Kirkwood (2001) has shown, in the case of the mouse life history, how within the framework of the disposable soma theory, there may be adaptive flexibility that results in fine tuning of the investment in maintenance. Such a flexibility is actually inherent to all life‐history traits, and the evolution of the optimal aging rate is just one example of such a trait.

## Evolvability

### Skulachev (1997)

Many advocates of programmed aging propose that a species which ages has a selection advantage because it evolves faster. For example, Skulachev (1997) wrote: *Death caused by aging clears the population of ancestors and frees space for progeny carrying new useful traits*. This is reminiscent of Weismann's idea, to which Skulachev referred. The difference is that, according to Skulachev, aging removes otherwise perfectly healthy individuals from the population in the hope that this is compensated by newborns carrying advantageous mutations. The problem with this idea is that most mutations are deleterious, so the next generation is not automatically better adapted. In the absence of aging (which must be assumed to be the original state, if the theory is not to be circular), it is not clear how Skulachev's death mechanism can actually target ‘ancestors’. Even if it could, removal of chronologically old individuals would be a process that selectively eliminates organisms of higher average fitness, because survival to higher ages would on average be a sign that the individual was endowed with a genotype of above‐average fitness. Skulachev did not propose any quantitative justification of his hypothesis, and on closer examination, it would seem that if one were to follow his argument, this would lead to the unintended consequence that aging actually delays evolution by selective removal of individuals with high intrinsic fitness.

### Goldsmith (2008)

Another hypothesis based on the idea that programmed death enhances ‘evolvability’ was put forward by Goldsmith (2008). He concentrates on sexually reproducing species and notes that sexual recombination generates a high genetic variation in the population that, according to Goldsmith, should speed up the evolution process. If aging shortens the mean lifespan, it also shortens the mean generation time and thus more genetic variants are ‘tested’ in the same time interval through sexual recombination. This hypothesis involves different selective forces (negative effect of lifespan shortening vs. hypothetical positive effect on genetic variation) acting in opposite directions and thus requires a mathematical model to visualize and understand its feasibility. Unfortunately, Goldsmith (2008) formulated his idea only verbally, but we decided to study its plausibility by developing an agent‐based computer simulation of its consequences.

Agent‐based computer simulations are a modern variant of cellular automata, where agents (here individuals of a population) live in a 2D or 3D environment and follow an arbitrarily complex set of rules. This kind of modelling is especially suitable to investigate population‐based questions, because it automatically handles spatial effects, which is important for studying phenomena based on kin or group selection. For our simulations, we used the excellent and freely available Java‐based software library MASON (Luke *et al*., 2005) that is designed for large‐scale and computationally demanding simulations. In our model, agents live in a 2D world (grid size 250 × 250) whose edges are cyclically connected and thus form the surface of a torus. In this model, the agents have three rules that allow them to move, reproduce and die. The movement rule creates a slow diffusion of agents simply to mimic the movement of animals in a landscape. Agents either move into free neighbouring fields or they exchange the position with a neighbouring agent. Historically, the four neighbours to the north, south, west and east form the so‐called von Neumann neighbourhood, and the eight neighbours including the four diagonal corners form the so‐called Moore neighbourhood. Reproduction depends on reaching an age of maturity and on a set of genes that can have values (alleles) ranging from 0 to 1, which contribute additively to the overall reproduction probability (fertility). As suggested by Goldsmith (2008), individuals reproduce sexually by selecting a mating partner from the (Moore) neighbourhood, with which they exchange genetic material; that is, each parent contributes 50% to the genes of the offspring. Finally, the agents can die because of an age‐independent environmental mortality, γ, or because they have reached a maximum lifespan at which they are killed (simulating programmed aging). Table 1 summarizes this set of rules.

**Table 1:**
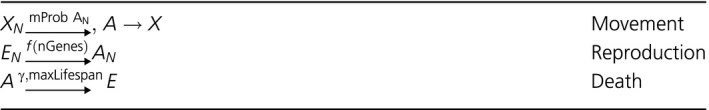
Set of rules that describe the behaviour of the agents in the simulation testing the evolvability idea of Goldsmith (2008). ‘*A*’ indicates the current agent, ‘*E*’ represents an empty field, and ‘*X*’ stands for any field content. Subscript ‘*N*’ indicates a field in the Moore neighbourhood; no subscript means the current field of the agent. The first rule states, for instance, that a field of the neighbourhood that can have an arbitrary content is filled, with a probability mProb, by the current agent. Furthermore, the current field of agent ‘*A*’ is filled by the entity ‘*X*’ (originating from *X*_*N*_)

In the agent‐based simulation, thousands of individuals compete with each other using the same set of rules, but with individual sets of genes governing reproduction. If this evolutionary selection process continues for thousands of time steps (each representing here 1 year), the alleles that contribute little to fertility will gradually be lost. To prevent this, we reverse the direction of selection in regular intervals (i.e. then small allele values lead to a high fertility) that can be specified in the graphical user interface (Fig. S1). We think that this is also in accordance with Goldsmith's idea, because his arguments imply that the greater genetic variability will speed up evolution especially under changing environmental conditions. The competition and selection occurring in the world of the agents offers also a natural way to study the evolution of a genetically programmed maximum lifespan. Each agent has not only its own inherited set of fertility genes, but also its own inherited maximum lifespan. However, when an offspring is generated, its maximum lifespan is ‘mutated’ such that it is a small amount (epsLifespan) larger or smaller than the lifespan of its parent. Selection then decides in which direction the programmed maximum lifespan of the population develops.

**Figure 2:**
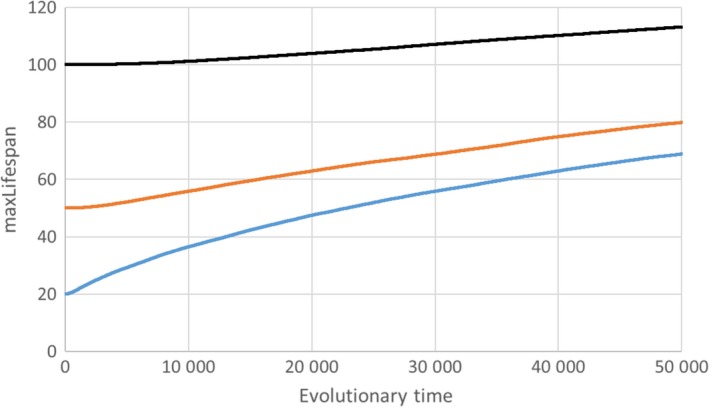
According to Goldsmith (2008), aging should evolve because it increases evolvability. However, our computer simulations show that selective forces always increase the maximum lifespan (shown here for three different starting values of maxLifespan). See main text for details. Used parameter values: Moore neighbourhood (8 neighbours), worldSize = 250 × 250, γ = 0.01, maturity = 17, epsLifespan = 0.1, nGenes = 30, selSwitchTime = 100 and mProb = 0.1.

Figure 2 shows typical results of our simulations. The diagram summarizes the results of three simulations that were started with all agents having a maximum lifespan of 20, 50 or 100 years. The genetically programmed maximum lifespan always increases over evolutionary time as a consequence of the selection process between individuals with lower and higher lifespan. We also performed simulations with different levels of environmental mortality, with different numbers of fertility genes and with different time intervals between a switch of the direction of selection (data not shown). We did not find any parameter combination that favoured a shortening of the maximum lifespan. As can be seen in the diagram, the lower the starting value of maxLifespan, the stronger is the increase over the 50 000 time steps. This is exactly what should be expected, because the force of natural selection declines with chronological age. Therefore, the increase in maxLifespan will slow down, but it will never stop.

So where is the flaw in Goldsmith's argument? Evolution is myopic such that there is no current reward for possible benefits in a far future. If a change in the environment (here a switch of the direction of selection) happens on a much longer time scale than the lifespan of individuals, there is no selection pressure to prepare for such a distant event. And if the environment changes on a time scale that is comparable to the species lifespan, then not enough time has passed to diminish the genetic variation of the population. In any case, a programmed limitation of the lifespan only has disadvantages (by killing agents) without any compensatory benefit.

As we have shown, Goldsmith's suggestion that programmed aging confers an evolutionary benefit by speeding up evolutionary progress fails within in its own terms. However, there is a further general objection to this hypothesis which is that the rate of production of progeny (and therefore the capacity to generate new adaptations) is determined by the time to maturity rather than the time to senescence and by the rate of genetic recombination and/or germ‐line mutation. Although there tends to be a correlation between time to maturity and lifespan, it is clear that selection will act more strongly on the former and that aging *per se* is unlikely to be of great potential consequence. Whether there is an optimal rate of genetic recombination and/or germ‐line mutation is an intriguing question that has been addressed within the extensive literature on evolution of sex, but it has little bearing on the question of programmed aging.

## Analytical models

### Libertini (1988)

**Figure 3:**
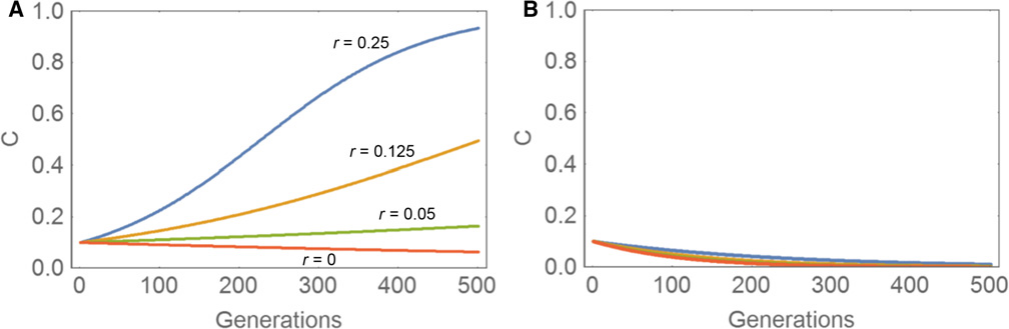
(A) Reproduction of Fig. 8 of Libertini (1988) showing the spread of a gene, *C*, causing aging for different degrees of relatedness to individuals in the neighbourhood. Used parameter values: *S* = 0.1, *S*′ = 0.001 and *V*_*C*_ = 0.7. (B) The same calculation as in A, but for more realistic parameter values. See main text for details. Used parameter values: *S* = 0.1, *S*′ = 0.02 and *V*_*C*_ = 0.7.

Libertini (1988) proposed that aging is programmed by focussing on the idea that a lifespan reduction via aging reduces the generation time. He argues that this would be of advantage for other beneficial genes in the population because the shorter the generation time, the faster is the spread of such genes. If a gene, *C*, that is causing aging (and is thus harmful to its carrier) has also an advantageous effect for other genetically related individuals, the harmful gene might be selected for, if the net selective advantage is positive. Libertini proposes that an individual which has died as a consequence of the action of gene *C* is replaced by a genetically related individual that also carries other advantageous genes, whose spreading speed is accelerated. The aging gene, *C*, would thus be some form of allelic hitchhiker that is co‐selected because it boosts the selection advantage of other genes that are beneficial. Thus, Libertini's argument is based on kin selection where a gene not only influences the fitness of its carrier, but also of relatives that carry the same gene. For kin selection, it is only necessary that relatives live closely together and because this condition is often fulfilled, Libertini raises here a valid argument for which he also developed a recurrence equation describing how the frequency of the aging gene, *C*, changes from generation to generation. The equation depends on the selection advantage, *S*, of the beneficial gene on which *C* hitchhikes, the selection disadvantage, *S*′, of the life shortening action of *C*, the degree of life shortening, *V*_*C*_, and the relatedness, *r*, of carriers of *C* with their neighbours. Based on that equation, Libertini calculated curves that seem to show that genes responsible for programmed aging can accumulate even with very low levels of relatedness (Fig. 3A).

$$C_{n+1}=\frac{C_{n}\cdot \left(1+r\cdot S\cdot \left(\frac{1}{V_{C}}-1\right)-S^{\prime }\right)}{1+C_{n}\cdot \left(r\cdot S\cdot \left(\frac{1}{V_{C}}-1\right)-S^{\prime }\right)}$$

However, Libertini's analysis has a serious problem. The lifespan reduction caused by aging, *V*_*C*_, is completely independent of the selection disadvantage, *S*′, that is caused by this phenotype. That means Libertini is free to choose arbitrary values for these two parameters. This allows him to maximize the positive effects (helping carriers of D by reducing the lifespan to *V*_*C*_) while at the same time minimizing the negative effects (selection disadvantage of early death) without any justification. If more realistic values are used, the aging gene *C* disappears (Fig. 3B).

## Spatial models

### Travis (2004)

Several of the ideas suggested to result in programmed aging involve concepts of kin selection that often invoke a spatial dimension. An explicit spatial model was described by Travis (2004) who developed his argument using agent‐based computer simulations. In his model, individuals follow rules for reproduction and death that are given in Table 2. Agents can die from an age‐independent mortality ‘*e*’ or they are killed (representing programmed aging) once they reach an age of death ‘*d*’. Reproduction generates an offspring in an empty field of the neighbourhood with a probability that declines with age, α, and is given by γ^α−1^. Travis then used the same evolutionary approach to find an optimal value for the programmed age of death, ‘*d*’, as we did in our model of the idea of Goldsmith (2008). The simulations showed that indeed ‘*d*’ either increased or decreased to approach a specific age of death. Travis concludes that an individual can increase its inclusive fitness by dying, if the space that is thus created is filled by a newborn kin that has a higher fertility. We completely agree with this explanation, but the critical point is that this is only true if fertility declines with age. The author realized this himself in his analysis: *If reproductive fitness does not decline with age, programmed death does not evolve. The model does not explain the evolution of senescence from a nonsenescing state*. But what then is the point of the whole idea? Obviously, a theory that proposes that aging is genetically programmed has to explain how such a program can evolve from a nonaging state.

**Table 2:**
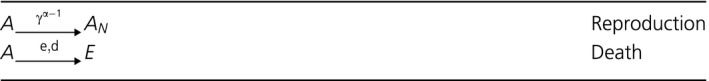
Set of rules that describe the behaviour of the agents according to Travis (2004). ‘*A*’ indicates the current agent, and ‘*E*’ represents an empty field. Subscript ‘*N*’ indicates a field in the Moore neighbourhood; no subscript means the current field of the agent. See main text for details

### Mitteldorf & Pepper (2009)

Another model that is based on the close spatial proximity of agents comes from Mitteldorf & Pepper (2009). In contrast to Travis (2004), fertility remains constant in this model and describes the probability that an agent chooses a random neighbour for reproduction. If the field is empty, an offspring is created; otherwise, reproduction fails. Also in this model, agents can die of an age‐independent background mortality, γ, or are killed at a programmed maximum age, maxLifespan. However, there is additionally a small probability, epiProb, that an agent starts an epidemic that instantly kills all members of the patch this agent belongs to (see Table 3). If maxLifespan is allowed to evolve, as described earlier, an optimal maxLifespan emerges that depends on the other model parameters. The authors therefore propose that senescence is an adaptation to limit the spread of disease.

**Table 3:**
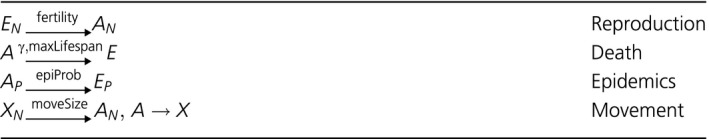
Set of rules that describe the behaviour of the agents according to Mitteldorf & Pepper (2009). ‘*A*’ indicates the current agent, ‘*E*’ represents an empty field, and ‘*X*’ stands for any field content. Subscript ‘*N*’ indicates a field in the Moore neighbourhood, a subscript ‘*P*’ represents agents belonging to the same patch, and no subscript means the current field of the agent. The movement rule is not part of Mitteldorf's model, but was added by us to explain how the original model works. See main text for details

**Figure 4:**
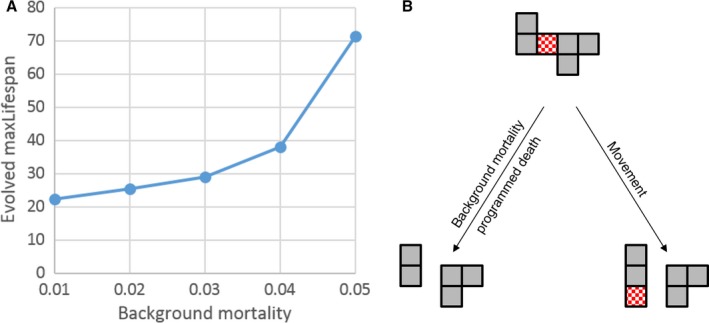
(A) Evolved values of maxLifespan as function of the background mortality, γ, in a simulation of the model of Mitteldorf & Pepper (2009). The higher the background mortality, the higher also the optimal value of the programmed age at which agents are killed. Parameters used: worldSize = 250 × 250, fertility = 0.1, epiProb = 1E‐5, epsLifespan = 0.1, Moore neighbourhood. (B) Background mortality and programmed death at maxLifespan are two ways how a patch of agents can split into two smaller patches, which mitigates the effects of an epidemic. But the same effect can also be achieved if agents could move (here the hatched agent). See main text for details.

We re‐implemented this model using the MASON library (Luke *et al*., 2005) and tried to reproduce Fig. 2 of Mitteldorf & Pepper (2009), which shows the dependence of evolved maxLifespan on the age‐independent background mortality. However, for the specified parameter settings, the population always died out after a short time span (Fig. S2). We suspect that there might be a mistake in the evolved maxLifespan (or fertility) of the original Figure because in all other diagrams of the authors, the shown maxLifespan is an order of magnitude lower. We therefore reduced fertility to 0.1 for our simulations and could then reproduce simulation results that are qualitatively identical with those of Mitteldorf (Fig. 4A). That means in an environment with low background mortality agents are killed young by programmed aging, while in a high‐risk environment programmed aging occurs very late in life. This is a surprising and counterintuitive result because normally a high environmental risk is associated with a fast aging rate, while long‐lived animals can be found in safe environments. Indeed, there is overwhelming experimental evidence for this correlation, as has been shown in field studies on opossums (Austad, 1993) and Daphnia (Dudycha & Tessier, 1999), in experimental evolution studies using Drosophila (Stearns *et al*., 2000) and in studies analysing survival data of multiple species of birds and mammals (Ricklefs, 1998) as well as poisonous vs. nonpoisonous animals (Blanco & Sherman, 2005). By contrast, there is only a single study based on guppies at variance with this trend (Reznick *et al*., 2004). The authors are aware of these data, but do not seem to regard them as counterevidence to their model.

**Figure 5:**
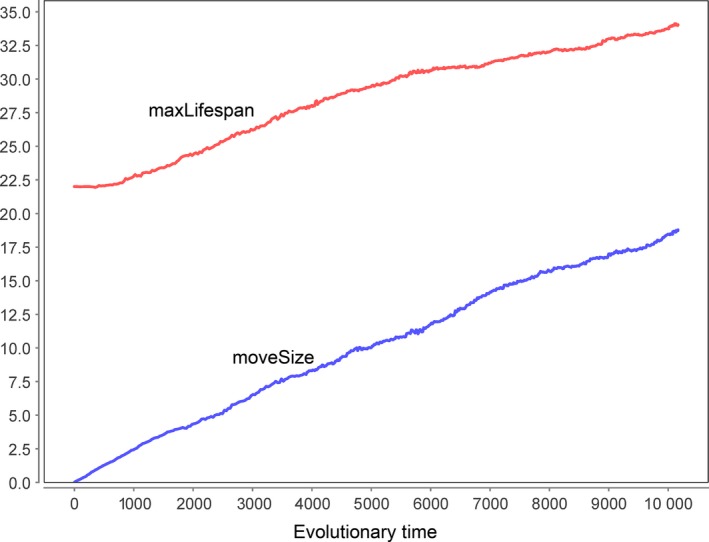
Simultaneous evolution of maxLifespan and moveSize in a simulation of the modified model of Mitteldorf & Pepper (2009). The simulation was started at the equilibrium point of maxLifespan for γ = 0.01 (left data point in Fig. 4A), with the addition that moveSize is allowed to evolve with a step size of epsMove = 0.1. Under these conditions, the system develops towards larger values for moveSize as well as maxLifespan. Other parameters: worldSize = 250 × 250, fertility = 0.1, epiProb = 1E‐5, epsLifespan = 0.1.

Closer inspection of the rules reveals why background mortality and programmed death are inversely correlated in this model. Both processes are a way to split large patches into smaller ones, thereby limiting the devastating effects of epidemics (Fig. 4B). Because there will be an optimal split rate that balances the positive effects (mitigating epidemics) against the negative effects (killing agents), it follows that a high background mortality is associated with few programmed deaths, and *vice versa*. To test this explanation, it is sufficient to introduce a single further rule that allows the agents to move (Table 3). As can be seen in Fig. 4B, this also leads to a splitting of large patches into smaller ones, but without killing agents. In the computer simulation shown in Fig. 5, maxLifespan as well as moveSize was allowed to evolve simultaneously. The system was started with a low background mortality (γ = 0.01), the corresponding optimal maxLifespan (left data point of Fig. 4A) and an initial moveSize of zero. As can be seen, the system develops a continuously increasing moveSize together with growing values for maxLifespan. This means that agents which reduce their patch size via movement are fitter than agents that do not move and split patches by programmed death. Such a behaviour could evolve only because the original set of rules given by Mitteldorf & Pepper (2009) did not allow for movement.

### Martins (2011)

A very interesting simulation was presented by Martins (2011), which is based on a simple set of rules (Table 4). In this model, reproduction can take place not only into empty neighbouring fields, but also into fields that are occupied by other agents. In that case, either the newborn or the existing agent dies with a probability that depends on the ‘fitness’ of both agents. Fitness is a property that an agent inherits from its parent, increased or decreased by an amount ‘*M*’ (representing mutations). Additionally, the fitness of each agent is decreased during each time step by an amount ‘*d*’, which is supposed to represent a changing environment that causes all agents to be slightly less adapted. Agents can die not only during a fight, but by being killed once they reach a certain ‘maxLifespan’, which represents programmed aging. Martins (2011) then performed several direct competition experiments between aging and nonaging agents and showed that there is an optimal value of ‘maxLifespan’, which he takes as indication that programmed aging can evolve.

**Table 4:**
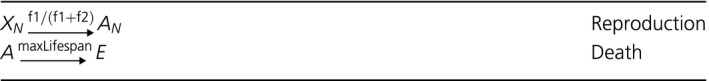
Set of rules that describe the behaviour of the agents according to Martins (2011). ‘*A*’ indicates the current agent, ‘*X*’ stands for any field content, and ‘*E*’ represents an empty field. Subscript ‘*N*’ indicates a field in the Moore neighbourhood; no subscript means the current field of the agent. See main text for details

**Figure 6:**
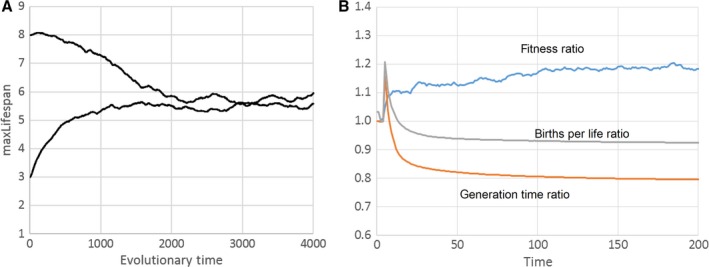
(A) Development of maxLifespan over evolutionary times in a simulation of the model of Martins (2011). The age at which individuals are killed increases or decreases to an optimum value that depends on the model settings. Parameters used: worldSize = 250 × 250, d = 0.01, *M* = 0.03, epsLifespan = 0.1. (B) Time course of relevant model variables during a competition experiment between agents without maximum lifespan (maxLifespan = 50) and those with a near‐optimal lifespan (maxLifespan = 5). For this, the 2D world was randomly initialized with 40% of aging and 40% of nonaging individuals. Other parameters as in (A) but with fixed values for maxLifespans (i.e. epsLifespan = 0). See main text for details.

Again, we re‐implemented this model using the MASON library (Luke *et al*., 2005) and performed simulations in which ‘maxLifespan’ was allowed to evolve as described earlier for the model of Goldsmith (2008). Figure 6A shows a typical simulation result, confirming that the evolutionarily optimal value of maxLifespan is around 5.5 under the used parameters (*d* = 0.01, *M* = 0.03). We also simulated direct competitions between agents with a near‐optimal maximum lifespan (maxLifespan = 5) and agents with an upper lifespan limit of 50. Because agents have a yearly mortality of roughly 50% (caused by fights with newborns), this is equivalent to a nonaging phenotype. Figure 6B shows the development of important model variables during the competition, which ended with the extermination of the nonaging species. The ratio of the fitness of the aging to the nonaging species quickly approaches values between 1.1 and 1.2, and Martins (2011) also noticed that the aging agents have a higher fitness. What might cause this higher fitness? The lower ratio of generation times points to the answer. Because aging agents have a shorter generation time, beneficial mutations (which are constantly generated by ‘*M*’) are spreading faster in such a population. Although there is also an associated cost in form of a reduced number of births per life, it seems that the increased fitness ratio outweighs this disadvantage. Taken together, the model of Martins (2011) is a practical implementation of the theoretical ideas of Libertini (1988).

**Figure 7:**
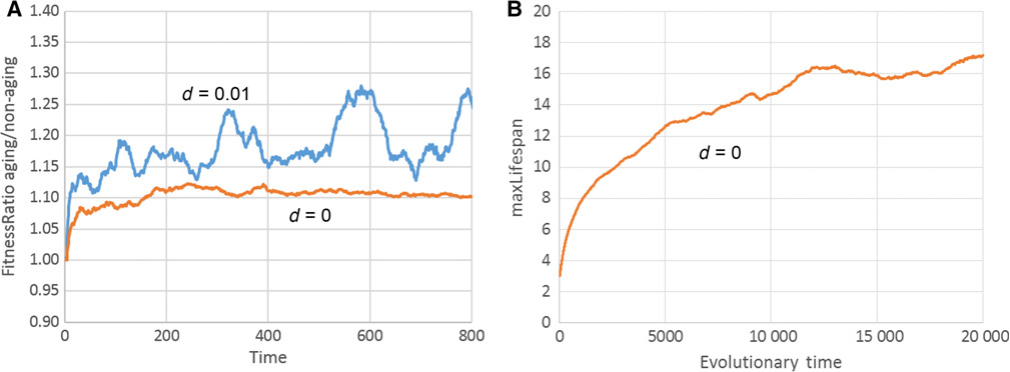
(A) Time course of the fitness ratio between aging and nonaging agents during a competition experiment. For *d* = 0, the aging phenotype still has a fitness advantage (ratio > 1), but it is consistently smaller than for *d* = 0.01. Under those conditions, the nonaging phenotype wins the contest. Parameters used: worldSize = 250 × 250, M = 0.03, maxLifespan = 50 vs. 5. (B) Development of maxLifespan over evolutionary times for *d* = 0. While for *d* > 0 maxLifespan approaches an equilibrium value (Fig. 6A), this is no longer the case if the constant decline of fitness is disabled. Parameters used: worldSize = 250 × 250, *d* = 0, *M* = 0.03, epsLifespan = 0.1.

The model does, however, rely on an unrealistically fast‐changing environment together with a constant influx of positive mutations. The fitness decline caused by ‘*d*’ is so rapid that environmental conditions change significantly within an individual's lifetime. Also, while an agent is alive, so many new positive mutations happen (caused by ‘*M*’) that after a few time steps, chronologically old agents have a lower fitness than newborns. We can rectify one of these problems by preventing a decrease in fitness (*d* = 0), while maintaining a positive value for new mutations (*M* > 0). Under these conditions, fitness will continuously increase, whereas it approaches a steady state if *d* > 0 (Fig. S3). But this is not a problem, because it is the relative fitness that decides the outcome of a fight between agents and not the absolute value. Interestingly, if direct competitions between an aging and nonaging genotype are performed with *d* = 0, it is now the nonaging phenotype that wins in >95% of the cases. Figure 7A compares the fitness ratio between aging and nonaging individuals during such a competition simulation for *d* = 0 and *d* = 0.01. In both cases, the aging phenotype has a higher fitness (ratio > 1), but for *d* = 0 this advantage is smaller than for *d* = 0.01. Taken together with the smaller number of births per life (Fig. 6B), this is sufficient that the Libertini mechanism no longer favours a programmed lifespan. This is also confirmed by allowing maxLifespan to evolve over time (Fig. 7B). Instead of approaching a fixed value as in Fig. 6A, the age of programmed death evolves to continuously increasing values, only slowed by the declining force of natural selection with chronological age.

**Figure 8:**
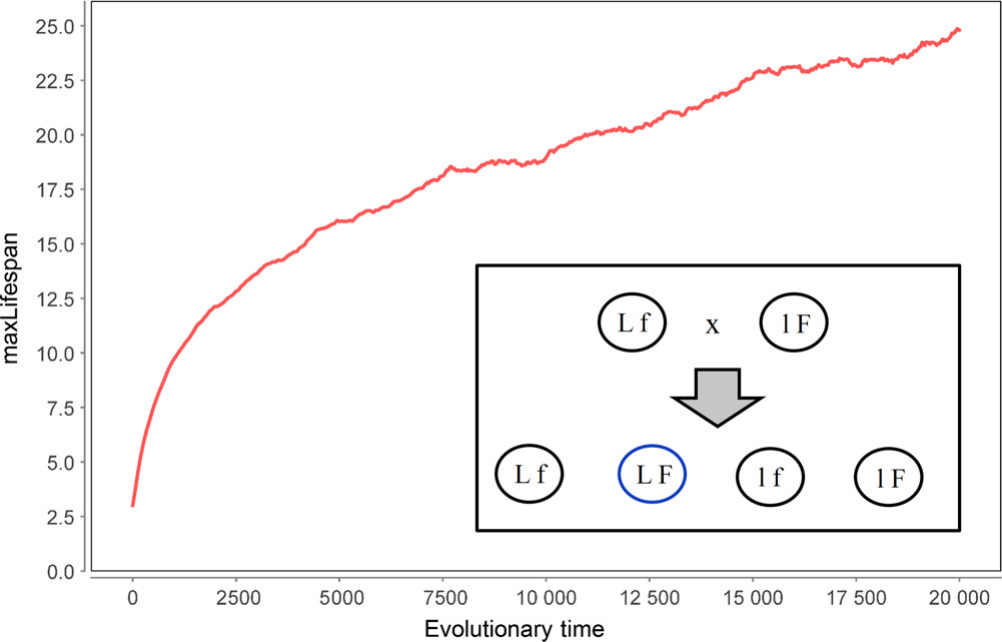
Development of maxLifespan over evolutionary times in a simulation of the model of Martins (2011) where agents reproduce sexually and inherit genes for maxLifespan, L, and fitness, F, from two parents. Using the same parameter settings as in Fig. 6A maxLifespan now increases without limit. The inset shows that if an agent with high maxLifespan and low fertility, Lf, breeds with an agent with low maxLifespan and high fertility, lF, all possible combinations can emerge including offspring with high lifespan and high fitness, LF. Parameters used: worldSize = 250 × 250, *d* = 0.01, *M* = 0.03, epsLifespan = 0.1.

The model of Martins (2011) has another, more fundamental, problem. The individuals in this model carry two properties (genes) that evolve over time, namely the maximum lifespan at which individuals are killed and the fitness that decides the outcome of a competition between individuals. The simulation as designed by Martins describes a population of clones that reproduce asexually. However, in reality, all higher animals reproduce sexually, a process during which offspring receive genes from both parents. Within life‐history theory generally, there is of course an extensive history dealing with the advantages and disadvantages of treating organisms as asexual or sexual. For analytic models in particular, it is often much easier to deal with the case of asexuality, but this can come at the cost of lack of biological realism. With a computational model, however, it is relatively straightforward to extend the model to account for sexual inheritance. We therefore wrote our simulation in such a way that agents can also reproduce sexually. In this case, a mating partner is chosen from the neighbourhood and the values for maxLifespan and fitness that the offspring receives are taken from one or the other individual. We then repeated the evolution of maxLifespan, which approached an optimal value of 5.5 under asexual conditions (Fig. 6A). However, using the more realistic sexual reproduction, the outcome is completely different (Fig. 8). Instead of approaching a stable equilibrium value, maxLifespan increased continuously without limit. The reason is simple. Because genes for maxLifespan and fitness can now originate from genetically different parents, it is possible that offspring are created which combine a long lifespan with a high fitness. These offspring are in the short term fitter than their parents and outcompete them. Thus, gene mixing caused by sexual reproduction leads to the emergence of ‘cheaters’ that enjoy a high fitness without paying for this via a short lifespan. Under those conditions, programmed aging does not evolve in the Martins model, even if there is a constant decline in fitness (*d* > 0).

### Mitteldorf & Martins (2014)

As mentioned above, the model of Martins (2011) assumes an unrealistically fast‐changing environment that leads to a loss of fitness within the lifetime of individual agents. To avoid this problem, Mitteldorf & Martins (2014) presented a new model with slightly modified rules. In this model, agents still inherit a fitness value from their parent, but this fitness no longer decreases at each time step. Furthermore, the ratio of positive to negative mutations affecting the fitness of offspring can be adjusted in this model variant, with positive mutations (that increase fitness by +1) occurring with a probability of 1/(1 + D) and negative mutations (that reduce fitness by −1) occurring with a probability of D/(1 + D). Thus if D = 0, there will be only positive mutations; if D = 1, the likelihood of negative and positive mutations is equal; and if D = ∞, only negative mutations happen.

Otherwise, the rules are similar to the earlier model in that agents can spawn offspring into empty as well as occupied neighbour cells and in that agents are killed once they reach a certain maximum lifespan. The only addition here is that agents can now also die because of an age‐independent mortality, ‘m’ (Table 5).

**Table 5:**
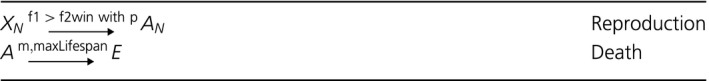
Set of rules that describe the behaviour of the agents according to Mitteldorf & Martins (2014). ‘*A*’ indicates the current agent, ‘*X*’ stands for any field content, and ‘*E*’ represents an empty field. Subscript ‘*N*’ indicates a field in the Moore neighbourhood; no subscript means the current field of the agent. See main text for details

**Figure 9:**
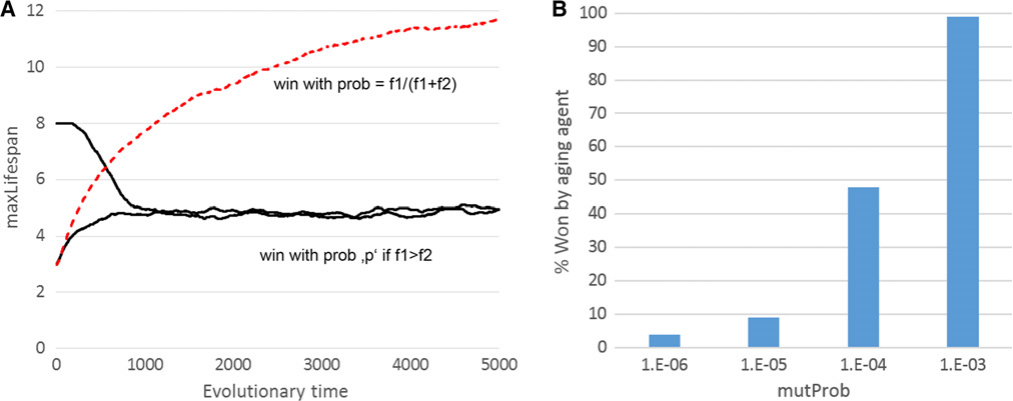
(A) Development of maxLifespan over evolutionary times according to Mitteldorf & Martins (2014) (continuous lines) and if the same rule for winning a fight between agents is used as in Martins (2011) (dashed line). In the first case, a specific maximum lifespan evolves; in the second case, maxLifespan increases without limit. Parameters used: worldSize = 250 × 250, *m* = 0.01, *D* = 1, *P* = 0.5, epsLifespan = 0.1. (B) Competition experiment between aging (maxLifespan = 5) and nonaging (maxLifespan = 5000) agents in an extended version of the model in which the mutation probability of the offspring fitness can be specified by mutProb. The lower this mutation probability, the less likely it is that the aging genotype wins against the nonaging genotype (each bar is based on 100 competitions). Parameters used: worldSize = 250 × 250, *m* = 0.01, *D* = 1, *P* = 0.5.

Figure 9A (continuous lines) shows that in this new model, the genetically programmed maximum lifespan also approaches a specific value if it is allowed to evolve freely. In the shown model simulation, a value of around five is approached either from below (starting from three) or from above (starting from eight). This is actually surprising because it was shown during the investigation of the earlier, similar model (Martins, 2011) that disabling the constant fitness decline leads to an unlimited increase in maxLifespan (Fig. 7B). Interestingly, the new model differs from the old in the way that the outcome of a combat between a newborn offspring and a resident agent is decided. In the old model, the probability to win was proportional to the fitness ratio of both agents, f1/(f1 + f2), while in the new model, a combat only commences if the fitness of the offspring is greater than the fitness of the neighbour and then the offspring wins with a probability of ‘*P*’ (which has a default value of 0.5). It is not clear why this new procedure has been used, because it is neither simpler nor more realistic than the old method. It is, however, clear that it has profound consequences for the outcome of the simulation. If the model of Mitteldorf & Martins (2014) is slightly modified so that it uses the old procedure, the evolution of maxLifespan leads again to continuously increasing ages (Fig. 9A, dashed line).

One aim of the new model was to make it more realistic by omitting the constant decline of fitness, which represented a rapidly changing environment in Martins’ original model. But the new model still has another unrealistic assumption; namely, that the mutation rate is unrealistically high. Every single offspring has its fitness mutated, either up or down. Because the total population size is roughly 60 000 individuals that reproduce at each time step, there are about 30 000 positive and 30 000 negative mutations each year, leading to a broth of fitness genotypes in the population (Fig. S4). The parameter ‘D’ that was introduced by the authors controls the ratio of positive to negative mutations, but the overall mutation rate remains the same. We therefore extended their model such that a fitness mutation happens only with a certain small probability, ‘mutProb’, and performed direct competition experiments between aging and nonaging individuals for different mutation probabilities. As can be seen in Fig. 9B, the success of genotypes with programmed death dwindles with decreasing ‘mutProb’. At a value of 10^−6^, corresponding to ca. 1 positive mutation per 30 time steps per population (Fig. S4), the aging genotype wins only 4% of the combats. The reason is that the mutation rate directly influences the percentage of carriers of beneficial mutations, whose spreading can be accelerated by a shorter lifespan (according to Libertini's equation). Although the total number of mutations per genome per generation is around one (Keightley *et al*., 2014), the number of beneficial mutations will be much smaller. And mutations with a large positive effect size will be especially rare. Thus, for more realistic mutation rates, the fraction of carriers becomes so small that programmed aging can no longer evolve.

**Figure 10:**
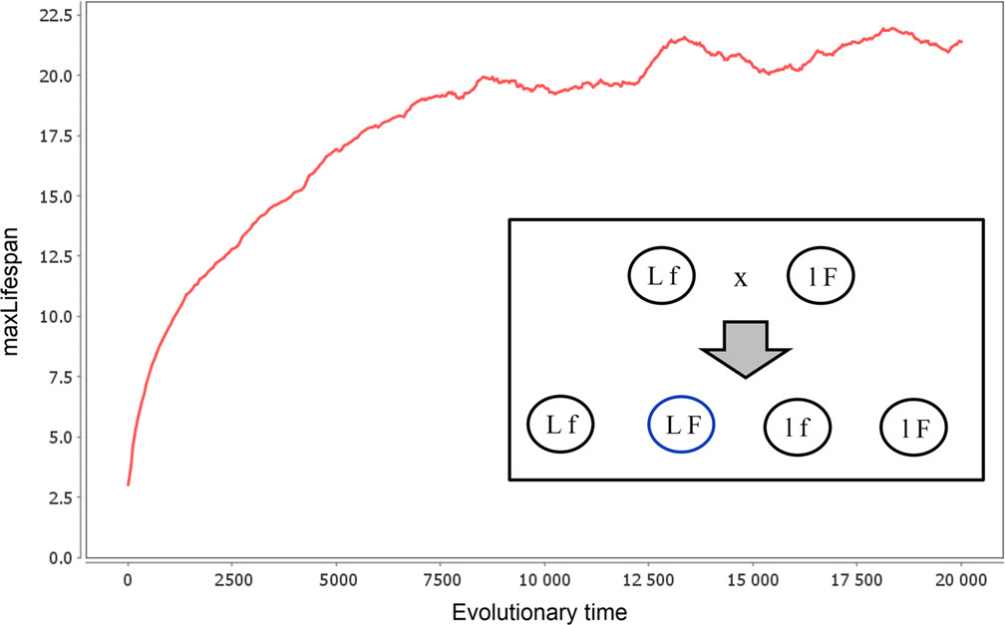
Development of maxLifespan over evolutionary times in a simulation of the model of Mitteldorf & Martins (2014) where agents reproduce sexually and inherit genes for maxLifespan, L, and fitness, F, from two parents. Using the same parameter settings as in Fig. 9A maxLifespan now increases without limit. The inset shows that if an agent with high maxLifespan and low fertility, Lf, breeds with an agent with low maxLifespan and high fertility, lF, all possible combination can emerge including offspring with high lifespan and high fitness, LF. Parameters used: worldSize = 250 × 250, *m* = 0.01, *D* = 1, *P* = 0.5, epsLifespan = 0.1, mutProb = 1.

Finally, we also investigated for this model how it responds to sexual reproduction. A mating partner is chosen from the neighbourhood and the offspring values (genes) for fitness and maxLifespan are randomly selected from one of the parents. The results, shown in Fig. 10, are very similar to the corresponding simulations of the original Martins model (Fig. 8). The age of programmed death rises without upper limit, only slowed by the declining force of natural selection. Because the number of survivors declines with increasing chronological age, the selection advantage of a further increase in maxLifespan declines, which manifests itself in a slower rise of maxLifespan. Thus, under sexual reproduction, also this model does not lead to the evolution of programmed aging.

### Werfel *et al*. (2015)

A very recent proposal that aging can be programmed comes from Werfel *et al*. (2015). The authors develop a spatial simulation that consists of two different types of agents, resources and consumers. Resources follow only a single rule, namely reproduction into free neighbours of a von Neumann neighbourhood with probability ‘*g*’ (Table 6). Consumers display a more complex behaviour and follow three rules. They reproduce by converting a resource in the neighbourhood into a new consumer with probability ‘*P*’, they can die by patch exhaustion (with probability ‘*v*’), leaving behind an empty patch, or they can undergo programmed death with probability ‘*q*’, leaving behind a resource. Computer simulations of Werfel *et al*. (2015) showed that if ‘*q*’ is allowed to evolve by increasing or decreasing its values in offspring by a small amount of epsilon, then the intrinsic mortality ‘*q*’ approaches a finite value, which is greater than zero. The authors took this as proof that programmed aging can evolve in spatial systems.

**Table 6:**
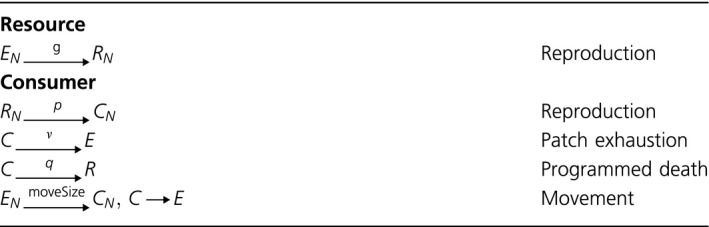
Set of rules that describe the behaviour of the agents according to Werfel *et al*. (2015). ‘*R*’ indicates a resource, ‘*C*’ stands for a consumer, and ‘*E*’ represents an empty field. Subscript ‘*N*’ indicates a field in the von Neumann neighbourhood; no subscript means the current field of the agent. The movement rule for consumers is not part of Werfel's model, but was added by us to explain how the original model works. See main text for details

**Figure 11:**
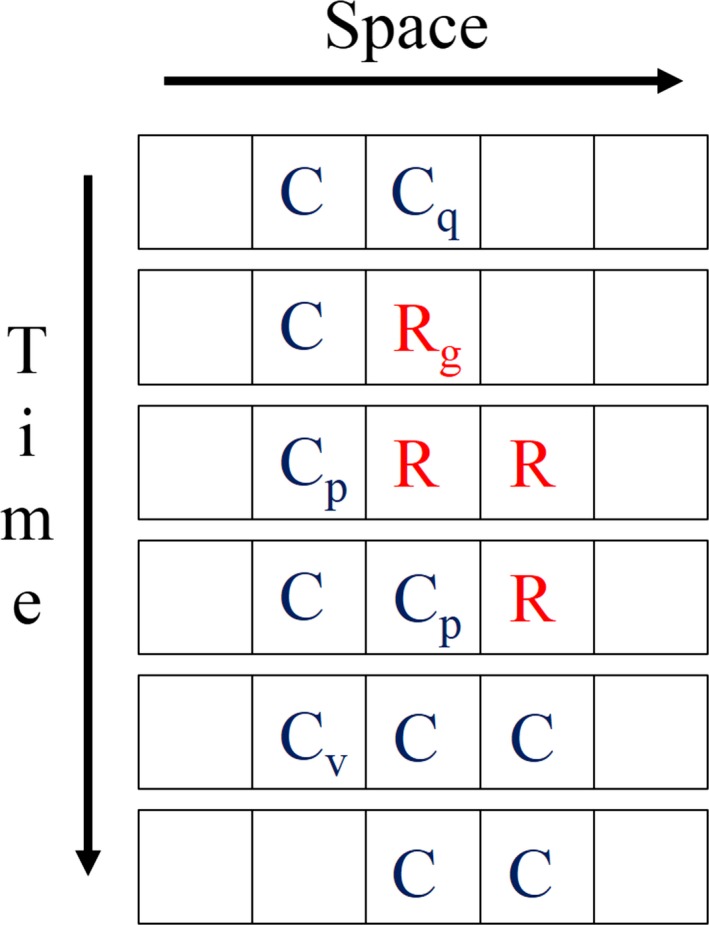
Sequence of events showing how a 1D patch of two consumers (top row) can move one grid element to the right (bottom row) in a series of five steps (shown on the vertical axis) according to the rules of Werfel *et al*. (2015). The event type is indicated by a subscript to the agent that performs the event; that is, for the first step, the right consumer undergoes programmed death (subscript *q*), so that a resource appears at that position.

Unfortunately, the authors did not provide any clear explanation why such an intrinsic mortality should evolve; that is, they did not explain the selection advantage that is conferred by programmed death. Therefore, we re‐implemented their model, again using the MASON library (Luke *et al*., 2005), and we were able to confirm that intrinsic mortality, *q*, evolves to an optimal value that depends on the model parameters (Fig. S5). However, by closely inspecting the set of rules for resource and consumer, it becomes apparent that the rule for programmed death allows consumers to move, a property that they otherwise do not possess. Figure 11 shows a sequence of five events that allows a pair of consumers (top row) to move one grid position to the right (bottom row) by temporarily creating some resource agents with the help of the rule for programmed death. First, the right consumer dies and leaves a resource. Then, this resource produces a second resource to the right. Next, there are two steps where consumers propagate by converting the neighbouring resource into a consumer. Finally, the leftmost consumer dies via patch exhaustion. Obviously, the ability to move is very helpful for isolated consumers, because it gives them a chance to reach distant resources and to reproduce by converting those resources into consumers of the same genotype.

**Figure 12:**
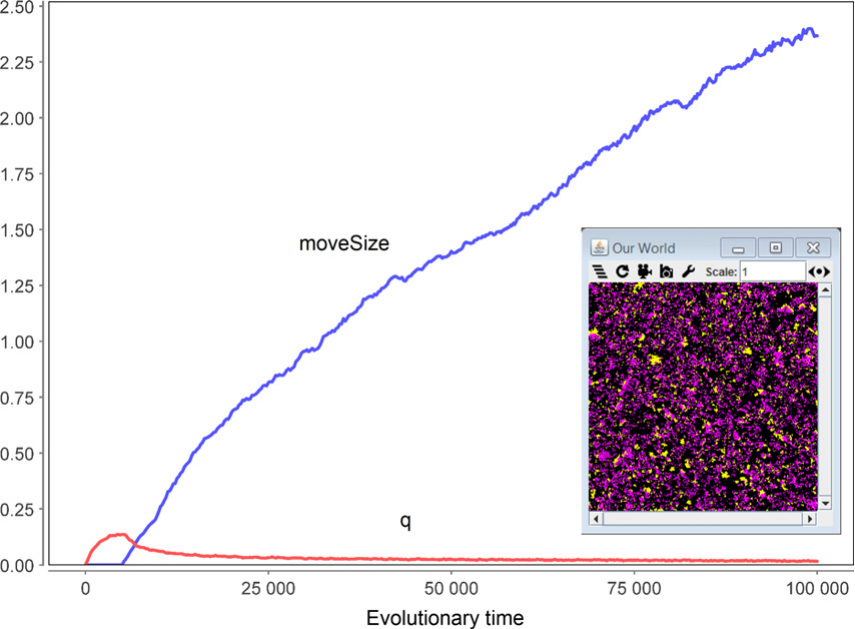
Evolution of the parameters ‘*q*’ and ‘moveSize’ in a simulation of the modified model of Werfel *et al*. (2015). For the first 5000 steps, only ‘*q*’ could evolve, approaching an equilibrium value of around 0.13. Then, also ‘moveSize’ was allowed to evolve starting from zero. As a consequence, ‘moveSize’ increased continuously, while ‘*q*’ dropped to ca. 0.02. The inset shows a snapshot of the simulation area with resources shown in yellow and consumers in magenta. Parameters used: worldSize = 250 × 250, *g* = 0.17, *P* = 0.9, *v* = 0.1, mutProb_q = 0.2, mutProb_m = 0.2, ε = 0.005.

To test this explanation, we introduced a new rule that allows a consumer to diffuse ‘moveSize’ grid elements in each simulation step (see Table 6). For instance, if moveSize = 1.7, there is a 70% chance to move 2 steps and a 30% chance to move 1 step. We performed a simulation in which we allowed ‘q’ to evolve for 5000 steps (starting from 0) after which it approached a steady‐state value of ca. 0.13. Then, also ‘moveSize’ was allowed to evolve and, as can be seen in Fig. 12, this caused a constant increase in ‘moveSize’, while at the same time ‘*q*’ decreased to around 0.02. Thus, given the possibility, the system prefers the nonlethal way of movement over the suicidal.

**Figure 13:**
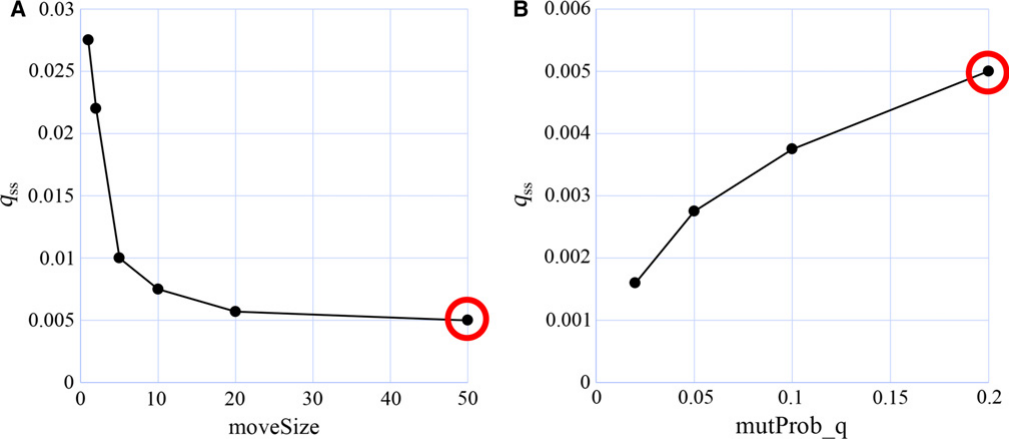
Simulation results of the modified model of Werfel *et al*. (2015). (A) If ‘moveSize’ is set to increasing values, ‘*q*’ evolves to progressively smaller steady‐state values. But even for moveSize = 50 a value of *q*_ss_ > 0 results. (B) If for moveSize = 50 the mutation probability of ‘*q*’ is reduced, also the corresponding steady‐state value of ‘*q*’ is reduced, indicating that the equilibrium value of ‘*q*’ in the population is only maintained via a mutation–selection balance. The red circle marks identical simulations. Parameters used: worldSize = 250 × 250, *g* = 0.17, *P* = 0.9, *v* = 0.1, ε = 0.005.

To test whether a large ‘moveSize’ can completely prevent the appearance of a finite value of ‘*q*’, simulations were performed in which ‘moveSize’ was fixed to different values and only ‘*q*’ was allowed to evolve to a steady‐state value. Figure 13A confirms that increasing values of ‘moveSize’ lead to declining steady‐state values of ‘*q*’, but even for extremely large values ‘*q*’ always remains greater than zero. Either it still provides some additional selection advantage by further increasing the ability to move (both methods to move are additive), or *q*_ss_ is maintained by a mutation–selection balance. In that case, *q* > 0 is not maintained in the population because it provides a selection advantage, but only because it is constantly re‐created via mutations. A way to test this is by changing the mutation rate. If *q*_ss_ is maintained via selection, the mutation rate should not influence the steady‐state value, while it should depend on it if a mutation–selection balance is at work. Therefore ‘moveSize’ was kept at 50 and the mutation rate was reduced below 0.2, which was the value used for the previous simulations. Figure 13B clearly shows that *q*_ss_ depends on the mutation rate and is thus maintained via a mutation–selection balance.

In summary, this confirms our suspicion that the results of Werfel *et al*. (2015) can be explained through a kin selection process in which lifespan is traded in to allow genetically related genotypes to move and thus reach resources that can be used for reproduction.

## Discussion

In recent years, there have been a number of publications claiming that the aging process is a genetically programmed trait that has some form of benefit in its own right. If this view were correct, it would be possible experimentally to identify the responsible genes and inhibit or block their action. This idea is, however, diametrically opposed to the mainstream view that aging has no benefit by its own and is therefore not genetically programmed. Because experimental strategies to understand and manipulate the aging process are strongly influenced by which of the two opinions is correct, we have undertaken here a comprehensive analysis of the specific proposals of programmed aging. On the principle that any challenge to the current orthodoxy should be taken seriously, our intention has been to see just how far the various hypotheses could go in building a convincing case for programmed aging.

We re‐implemented computational models (Mitteldorf & Pepper, 2009; Martins, 2011; Mitteldorf & Martins, 2014; Werfel *et al*., 2015), developed new computational models (Goldsmith, 2008) and analysed mathematical equations (Libertini, 1988; Goldsmith, 2012, 2013). The results fall into three classes. Either the ideas did not work because they are mathematically or conceptually wrong (Travis, 2004; Goldsmith, 2008, 2012, 2013), or programmed death did evolve in the models but only because it granted individuals the ability to move (Mitteldorf & Pepper, 2009; Werfel *et al*., 2015), or programmed death did evolve because it shortened the generation time (Martins, 2011; Mitteldorf & Martins, 2014) and thus accelerated the spread of beneficial mutations as originally described by Libertini (1988).

The last case is the most interesting, but it is, nevertheless, flawed. It only works if an unrealistically fast‐changing environment (Fig. 7B) or an unrealistically high number of beneficial mutations (Fig. 9B) are assumed. Furthermore and most importantly, it only works for an asexual mode of reproduction. If sexual reproduction is introduced into the models, the idea of Libertini that programmed aging speeds up the spread of advantageous mutations by shortening the generation time does not work at all (Figs 8 and 10). The reason is that sexual reproduction enables the generation of offspring that combine the nonaging genotype of one parent with the beneficial mutation(s) found in the other parent. The presence of such ‘cheater’ offspring does not allow the evolution of agents with programmed aging.

In summary, all of the studied proposals for the evolution of programmed aging are flawed. Indeed, an even stronger objection to the idea that aging is driven by a genetic programme is the empirical fact that among the many thousands of individual animals that have been subjected to mutational screens in the search for genes that confer increased lifespan, none has yet been found that abolishes aging altogether (Kirkwood & Melov, 2011). If such aging genes existed as would be implied by programmed aging, they would be susceptible to inactivation by mutation. This strengthens the case to put the emphasis firmly on the logically valid explanations for the evolution of aging based on the declining force of natural selection with chronological age, as recognized more than 60 years ago by Medawar (1952) and as developed further by Hamilton (1966) and Charlesworth (1980). The three nonprogrammed theories that are based on this insight [mutation accumulation (Medawar, 1952), antagonistic pleiotropy (Williams, 1957) and disposable soma (Kirkwood, 1977)] are not mutually exclusive. There is much yet to be understood about the details of why and how the diverse life histories of extant species have evolved, and there are plenty of theoretical and experimental challenges to be met. As we observed earlier, there is a natural attraction to the idea that aging is programmed, because developmental programming underpins so much else in life. Yet aging truly is different from development, even though developmental factors can influence the trajectory of events that play out during the aging process. To interpret the full complexity of the molecular regulation of aging via the nonprogrammed theories of its evolution may be difficult, but to do it using demonstrably flawed concepts of programmed aging will be impossible.

## Funding

No funding information provided.

## Conflict of interest

None declared.

## Supporting information


**Data S1.** Description of the program code used to generate the different simulations.

**Data S2.** Java program for the investigation of the idea of Goldsmith (2008).

**Data S3.** Java program for the investigation of the idea of Mitteldorf and Pepper (2009).

**Data S4.** Java program for the investigation of the idea of Martins (2011).

**Data S5.** Java program for the investigation of the idea of Mitteldorf and Martins (2014).

**Data S6.** Java program for the investigation of the idea of Werfel (2015).

**Fig. S1** Agent‐based simulation of the idea of Goldsmith (2008) using the MASON library (Luke *et al*., 2005).

**Fig. S2** Agent‐based simulation of the idea of Mitteldorf & Pepper (2009) using the MASON library (Luke *et al*., 2005).

**Fig. S3** Agent‐based simulation of the idea of Martins (2011) using the MASON library (Luke *et al*., 2005).

**Fig. S4** Agent‐based simulation of the idea of Mitteldorf & Martins (2014) using the MASON library (Luke *et al*., 2005).

**Fig. S5** Agent‐based computer simulations of the idea of Werfel *et al*. (2015) using the MASON library (Luke *et al*., 2005) show that the probability to suffer programmed death, q, approaches over evolutionary times a certain optimal value that depends on the model parameters.
