# Supplementary material for: Can aging be programmed? A critical literature review
Source: Aging Cell. 2016 Aug 17;15(6):986–98. doi: 10.1111/acel.12510 (PMC6398523; doi:10.1111/acel.12510)
Supplement: Supplementary file 1 — Data S1. Description of the program code used to generate the different simulations. [file ACEL-15-986-s001.docx]

## Software

We developed all simulations in Java using the software library MASON (<http://cs.gmu.edu/~eclab/projects/mason/>). Since we regard it as important that simulation results can be reproduced, we make our programs available as executable as well as source code.

**EvolvabilityWithGUI.jar**

This file contains the code for the investigation of the idea of [Goldsmith (2008)](#_ENREF_13). On all computers with a Java installation the graphical user interface can be started with:
java –jar EvolvabilityWithGUI.jar
The GUI contains four tabs, with “Model” being the most important since it allows to view and set the simulation parameters. Hovering the mouse pointer over the parameters brings up a short tooltip describing the parameter. The simulation itself can then be started with the start, pause and stop buttons at the bottom of the GUI.
The jar file also contains all the source code in the folder src, which can be extracted with jar xvf EvolvabilityWithGUI.jar. In Evolvabilty.java the simulation environment is initialised and agents are placed on the 2D world. Agent.java contains the code that controls the behaviour of the agent. During each time step of the simulation the method step() is called, which performs the different actions of the agent.

**Mitteldorf09WithGUI.jar**

This file contains the code and source for the investigation of the idea of [Mitteldorf and Pepper (2009)](#_ENREF_30). The structure and usage is the same for all our jar files.

**MartinsWithGUI.jar**

This file contains the code and source for the investigation of the idea of [Martins (2011)](#_ENREF_26).

**MiMa14WithGUI.jar**

This file contains the code and source for the investigation of the idea of [Mitteldorf and Martins (2014)](#_ENREF_29).

**Werfel15withGUI.jar**

This file contains the code and source for the investigation of the idea of [Werfel *et al.* (2015)](#_ENREF_39).
