# Supplementary figures and images for: Can aging be programmed? A critical literature review
Source: Aging Cell. 2016 Aug 17;15(6):986–98. doi: 10.1111/acel.12510 (PMC6398523; doi:10.1111/acel.12510)

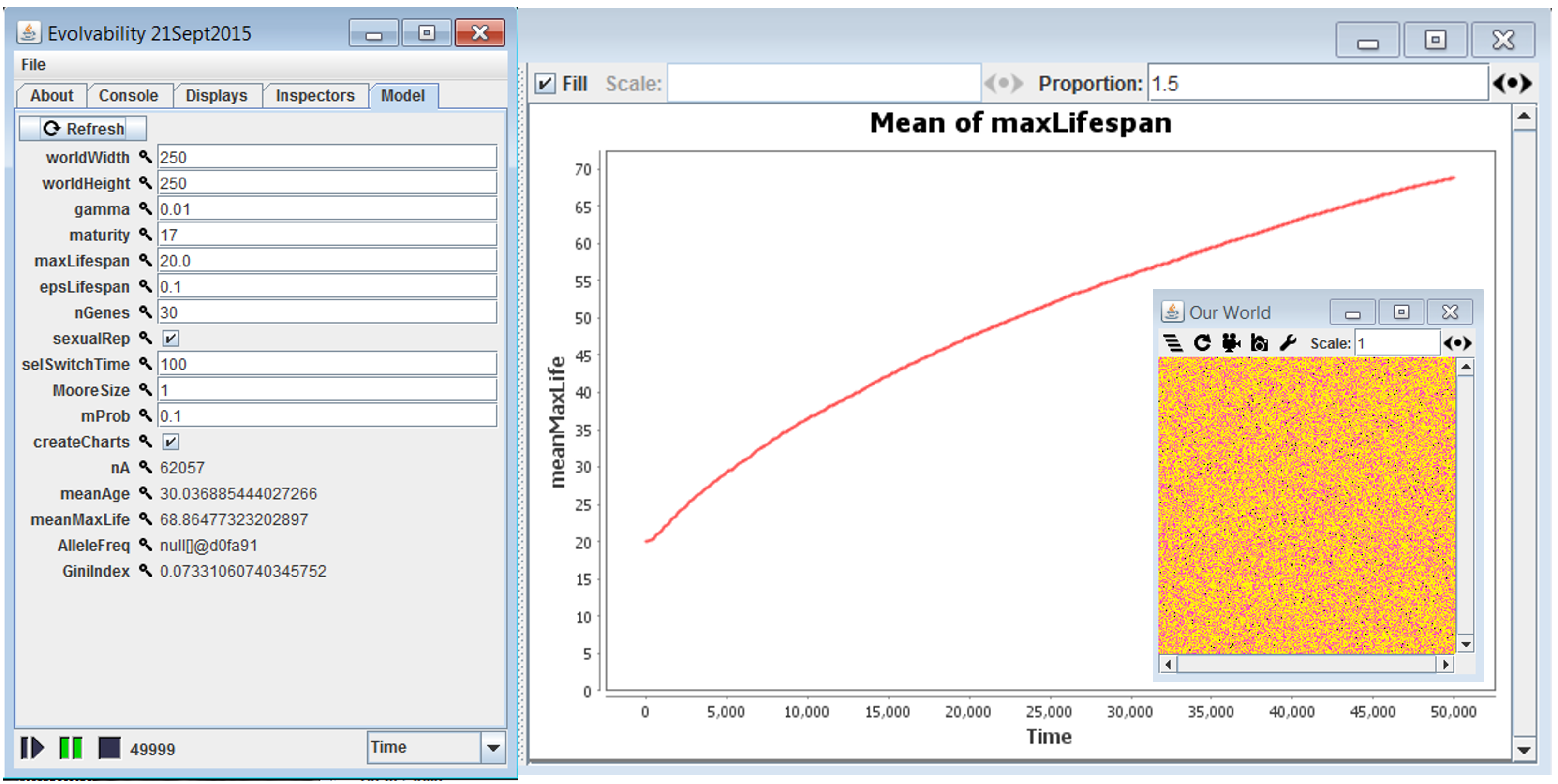

Supplement: Supplementary file 7 — Fig. S1 Agent‐based simulation of the idea of Goldsmith (2008) using the MASON library (Luke et al., 2005). [file ACEL-15-986-s007.tif]

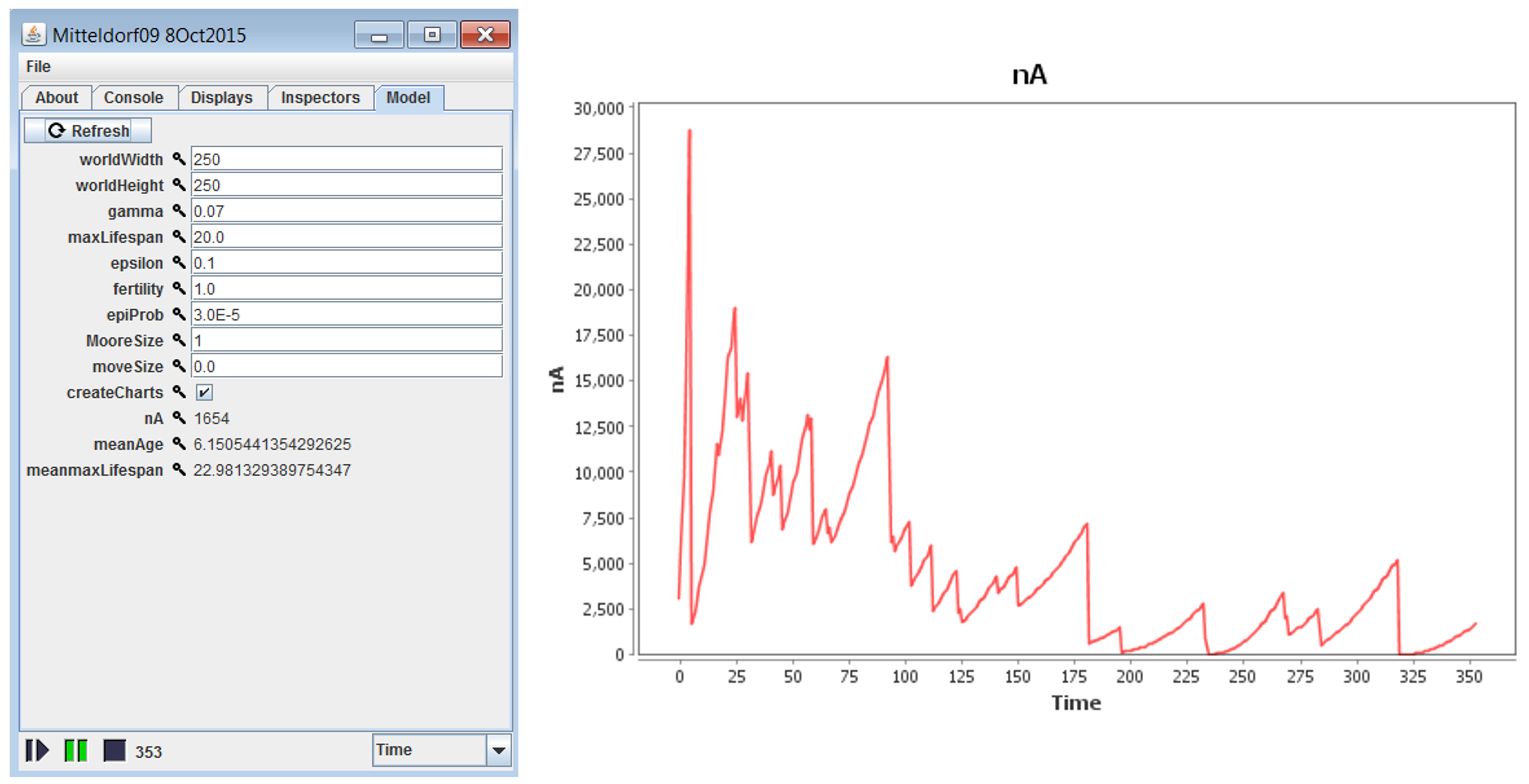

Supplement: Supplementary file 8 — Fig. S2 Agent‐based simulation of the idea of Mitteldorf & Pepper (2009) using the MASON library (Luke et al., 2005). [file ACEL-15-986-s008.tif]

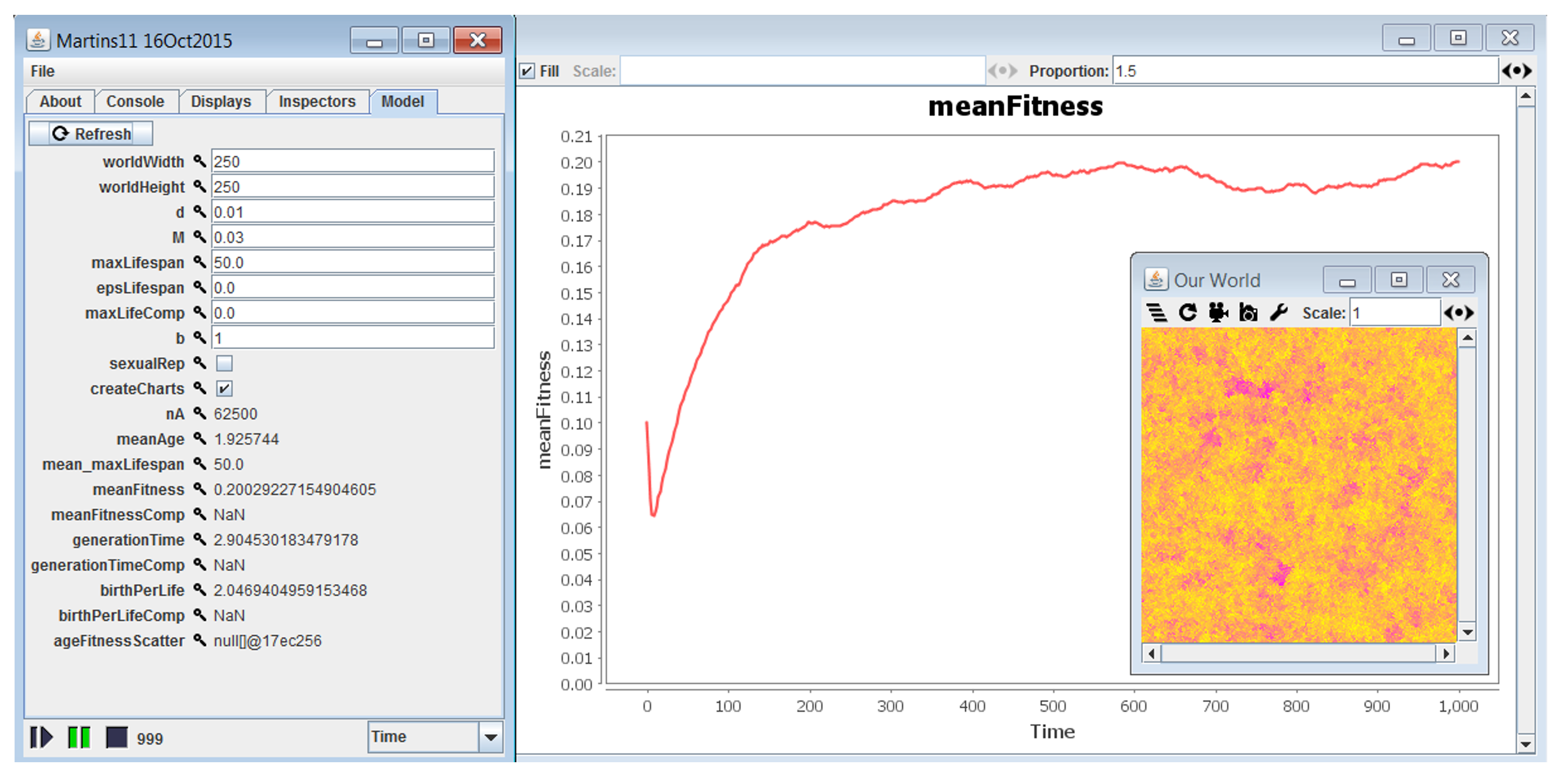

Supplement: Supplementary file 9 — Fig. S3 Agent‐based simulation of the idea of Martins (2011) using the MASON library (Luke et al., 2005). [file ACEL-15-986-s009.tif]

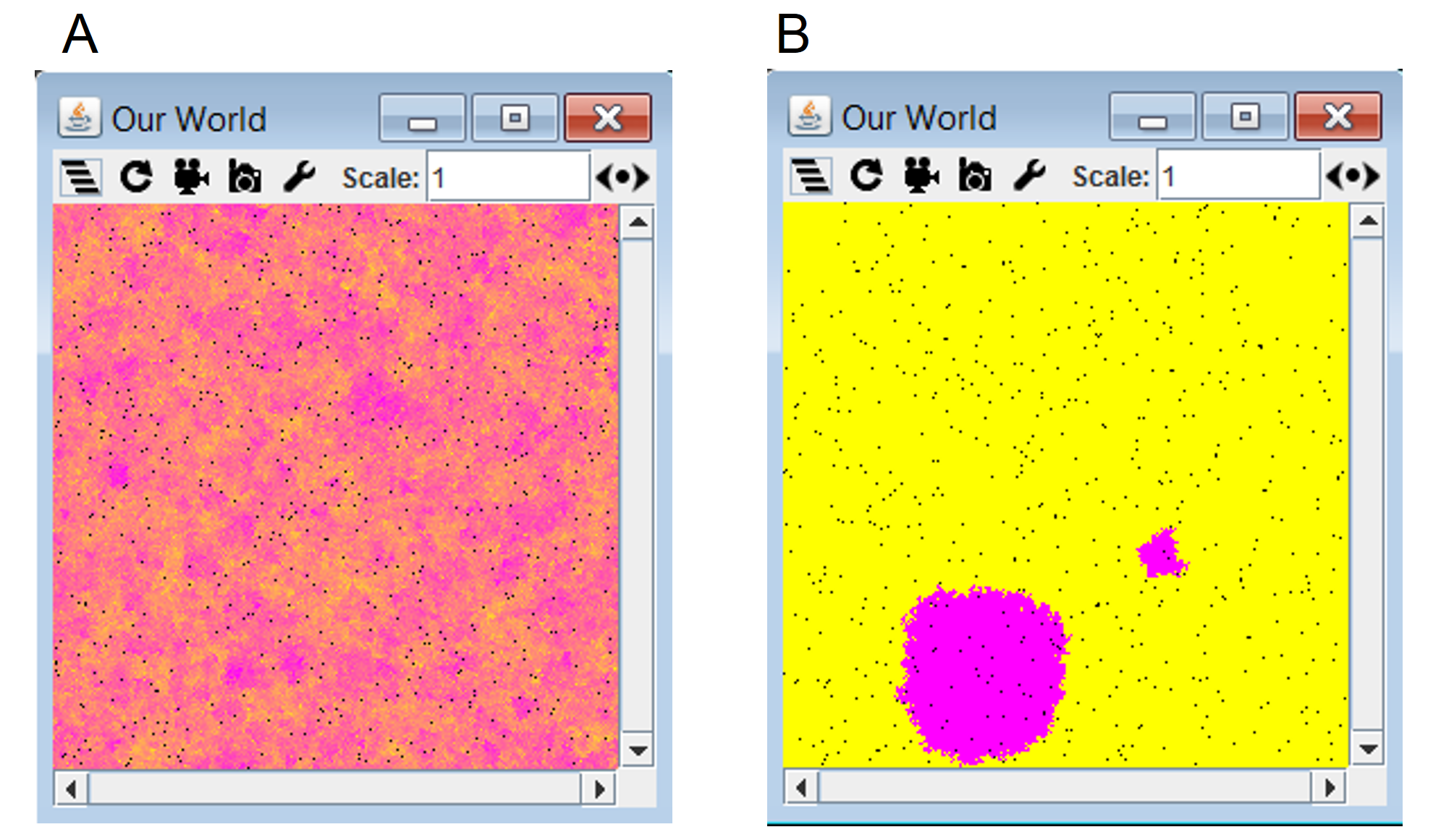

Supplement: Supplementary file 10 — Fig. S4 Agent‐based simulation of the idea of Mitteldorf & Martins (2014) using the MASON library (Luke et al., 2005). [file ACEL-15-986-s010.tif]

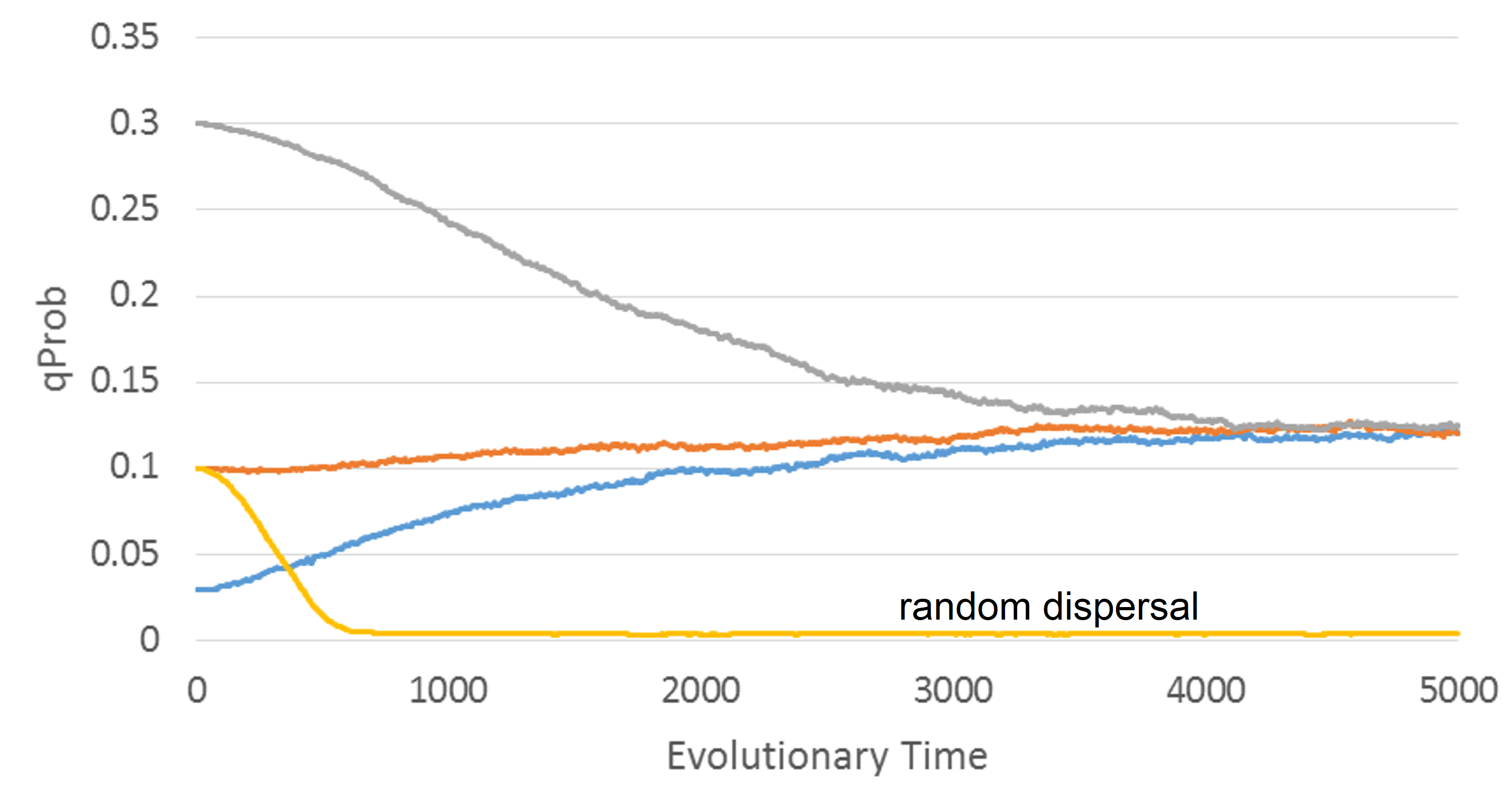

Supplement: Supplementary file 11 — Fig. S5 Agent‐based computer simulations of the idea of Werfel et al. (2015) using the MASON library (Luke et al., 2005) show that the probability to suffer programmed death, q, approaches over evolutionary times a certain optimal value that depends on the model parameters. [file ACEL-15-986-s011.tif]
