# Supplementary material for: Can aging be programmed? A critical literature review
Source: Aging Cell. 2016 Aug 17;15(6):986–98. doi: 10.1111/acel.12510 (PMC6398523; doi:10.1111/acel.12510)
Supplement: Supplementary file 12 [file ACEL-15-986-s012.docx]

Software

We developed all simulations in Java using the software library MASON (http://cs.gmu.edu/~eclab/projects/mason/). Because we regard it as important that simulation results can be reproduced, we make our programs available as executable as well as source code.

**EvolvabilityWithGUI.jar**

This file contains the code for the investigation of the idea of Goldsmith (2008). On all computers with a Java installation, the graphical user interface can be started with: java –jar EvolvabilityWithGUI.jar
The GUI contains four tabs, with ‘model’ being the most important because it allows to view and set the simulation parameters. Hovering the mouse pointer over the parameters brings up a short tooltip describing the parameter. The simulation itself can then be started with the start, pause and stop buttons at the bottom of the GUI.
The jar file also contains all the source codes in the folder src, which can be extracted with jar xvf EvolvabilityWithGUI.jar. In Evolvabilty.java, the simulation environment is initialized and agents are placed on the 2D world. Agent.java contains the code that controls the behaviour of the agent. During each time step of the simulation, the method step() is called, which performs the different actions of the agent.

**Mitteldorf09WithGUI.jar**

This file contains the code and source for the investigation of the idea of Mitteldorf and Pepper (2009). The structure and usage is the same for all our jar files.

**MartinsWithGUI.jar**

This file contains the code and source for the investigation of the idea of Martins (2011).

**MiMa14WithGUI.jar**

This file contains the code and source for the investigation of the idea of Mitteldorf and Martins (2014).

**Werfel15withGUI.jar**

This file contains the code and source for the investigation of the idea of Werfel *et al.* (2015).

Fig. S 1: Agent-based simulation of the idea of Goldsmith (2008) using the MASON library (Luke et al., 2005). The model parameters can be specified via a GUI (left side), which also allows to start and stop the simulation. Model properties can be displayed in separate windows (right) that are updated automatically. The simulation results depend on the actions of the agents in the simulated 2D environment (small inset with agents coloured according to age).

Fig. S 2: Agent-based simulation of the idea of Mitteldorf and Pepper (2009) using the MASON library (Luke et al., 2005). This simulation aims at reproducing a data point of Fig. 2 in the original publication, but for the specified parameters the simulation always collapses after a short time. This snapshot is shortly before the population dies out completely.

Fig. S 3: Agent-based simulation of the idea of Martins (2011) using the MASON library (Luke et al., 2005). This simulation shows that the mean fitness of the population always approaches an equilibrium value irrespectively of the values of ‘d’ and ‘M’. If ‘M’ is large enough to balance the fitness loss caused by ‘d’, fitness initially rises but hereby the selection advantage, that a new positive mutant has compared to the population mean, shrinks. And a smaller selection advantage causes a slower spread of positive mutants up to the point that the fitness gain caused by beneficial mutants is exactly balanced by the loss caused by ‘d’. The inset shows the 2D world filled with agents that are coloured based on their fitness.

Fig. S 4: Agent-based simulation of the idea of Mitteldorf and Martins (2014) using the MASON library (Luke et al., 2005). These simulations show the 2D world of agents for different mutation probabilities of the fitness property. Colours indicate the fitness of the agents. A) The normal mutation rate as specified by the model (mutProb = 1) leads to a broth of agents with different fitness values. B) If the mutation rate is reduced to more realistic values (mutProb = 10^-6^), mutants occur much more rarely and expand then into the surrounding population. Parameters used: worldSize = 250x250, m = 0.01, D = 1, p = 0.5, maxLifespan = 5000, epsLifespan = 0.

Fig. S 5: Agent-based computer simulations of the idea of Werfel et al. (2015) using the MASON library (Luke et al., 2005) show that the probability to suffer programmed death, q, approaches over evolutionary times a certain optimal value that depends on the model parameters. This effect is shown here for three different starting values of q. This phenomenon is clearly connected to the spatial structure of the model, because random dispersal of offspring leads to a rapid drop of programmed death. Parameters used: worldSize = 250x250, g = 0.2, p = 0.9, v = 0.1, mutProb_q = 0.2, epsilon = 0.005.
